# Phosphatidylserine Ameliorates Neurodegenerative Symptoms and Enhances Axonal Transport in a Mouse Model of Familial Dysautonomia

**DOI:** 10.1371/journal.pgen.1006486

**Published:** 2016-12-20

**Authors:** Shiran Naftelberg, Ziv Abramovitch, Shani Gluska, Sivan Yannai, Yuvraj Joshi, Maya Donyo, Keren Ben-Yaakov, Tal Gradus, Jonathan Zonszain, Chen Farhy, Ruth Ashery-Padan, Eran Perlson, Gil Ast

**Affiliations:** 1 Department of Human Molecular Genetics and Biochemestry. Sackler Faculty of Medicine, Tel Aviv University, Tel Aviv, Israel; 2 Department of Physiology and Pharmacology, Sackler Faculty of Medicine, Tel Aviv University, Tel Aviv, Israel; Stanford University School of Medicine, UNITED STATES

## Abstract

Familial Dysautonomia (FD) is a neurodegenerative disease in which aberrant tissue-specific splicing of *IKBKAP* exon 20 leads to reduction of IKAP protein levels in neuronal tissues. Here we generated a conditional knockout (CKO) mouse in which exon 20 of *IKBKAP* is deleted in the nervous system. The CKO FD mice exhibit developmental delays, sensory abnormalities, and less organized dorsal root ganglia (DRGs) with attenuated axons compared to wild-type mice. Furthermore, the CKO FD DRGs show elevated HDAC6 levels, reduced acetylated α-tubulin, unstable microtubules, and impairment of axonal retrograde transport of nerve growth factor (NGF). These abnormalities in DRG properties underlie neuronal degeneration and FD symptoms. Phosphatidylserine treatment decreased HDAC6 levels and thus increased acetylation of α-tubulin. Further PS treatment resulted in recovery of axonal outgrowth and enhanced retrograde axonal transport by decreasing histone deacetylase 6 (HDAC6) levels and thus increasing acetylation of α-tubulin levels. Thus, we have identified the molecular pathway that leads to neurodegeneration in FD and have demonstrated that phosphatidylserine treatment has the potential to slow progression of neurodegeneration.

## Author Summary

We create a novel FD mouse model, in which exon 20 of *IKBKAP* was deleted in the nervous system, to study the role of IKAP in the neurodegeneration process. The lack of *IKBKAP* exon 20 impaired retrograde nerve growth factor (NGF) transport and axonal outgrowth. Reduction of IKAP levels resulted in elevated HDAC6 levels and thus reduced acetylated α-tubulin levels. Phosphatidylserine down-regulated HDAC6 levels, furthermore phosphatidylserine treatment facilitated axonal transport and stabilized microtubules. In brief: Naftelberg et al. identify the molecular pathway leading to neurodegeneration using a mouse model of familial dysautonomia and suggest that phosphatidylserine acts as an HDAC6 inhibitor to improve neurologic function.

## Introduction

Familial Dysautonomia (FD) is an autosomal recessive congenital neuropathy that occurs almost exclusively in the Ashkenazi Jewish population with a remarkably high carrier frequency ranging from 1 in 18 (in those of Polish descent) to 1 in 32 [1]. Individuals with FD suffer from a variety of symptoms including vomiting crises, pneumonia, ataxia, difficulty swallowing, gastrointestinal and cardiovascular dysfunction, and short life spans [2–6]. Previous work discovered that the underlying genetic cause of FD is a point mutation in the *IKBKAP* gene, which encodes the IκB kinase complex-associated protein (IKAP) [7,8]. A transition from T to C at position 6 of the 5’ splice site of *IKBKAP* intron 20 [8] alters the splicing pattern of the *IKBKAP* gene in a tissue-specific manner: There is a shift from constitutive inclusion of exon 20 to alternative splicing in all tissues, and in the nerve tissues this exon is predominantly skipped [9]. As a result of the exon 20 skipping, a premature stop codon is generated. No truncated protein has been detected in tissues of FD patients [8,10,11]; however, there is a considerable reduction in full-length IKAP protein expression in the nervous systems of FD patients [8,11].

FD patients exhibit abnormal development and progressive depletion of unmyelinated sensory and autonomic neurons [12–16]. Although the central neuropathology in FD is poorly defined, recent MRI studies indicate that FD patients have abnormal proportions of white matter, decreased optic radiation, and cerebellar microstructural alterations compared to healthy volunteers [17]. The lack of IKAP also results in reduced size and numbers of dorsal-root ganglion (DRG) and sympathetic ganglion (SG) neurons [13,18–20]. DRGs are highly polarized cells that depend on active intracellular transport mechanisms in order to survive and properly function. Postsynaptic targets release neurotrophins like nerve growth factor (NGF) that move in a retrograde fashion along the axon to the soma to evoke changes in gene expression [21,22]. Although alterations in this axonal transport process is linked to many neurodegenerative diseases and may be involved in FD [19,23,24], the molecular mechanism that underlies the alterations in transport is unknown.

IKAP has been studied extensively, and findings point to an unexpected diversity of IKAP actions. Early findings indicated that IKAP (also known as ELP1) is a subunit of the Elongator complex, important for RNA polymerase II transcription elongation in the nucleus and for histone acetylation [25–29]. As IKAP co-localizes and purifies with cytoplasmic proteins [30–32], it has been suggested that IKAP functions in tRNA modification [33–35], exocytosis [36], cell adhesion and migration, microtubule organization [20,32,37,38], p53 activation [39], and c-Jun N-terminal kinase (JNK) signaling pathway regulation [19,23,31]. Recent studies focused on IKAP function in neurons suggest that IKAP influences oligodendrocyte differentiation and myelin formation [40,41], is crucial for vascular and peripheral neural development during embryogenesis [20,42,43], regulates NGF signaling, and distributes target innervations [19,20]. Deletion of IKAP in migrating neural crest further documented a key role for IKAP in DRG progenitors for correct timing of neurogenesis and survival of TrkA^+^ nociceptors and thermoreceptors [44]. These findings demonstrate that IKAP plays an essential role during neuronal development. FD patients exhibit progressive DRG neurodegeneration, but the underlying molecular mechanism by which IKAP deficiency result in this degeneration has still not been established.

**Fig 1:**
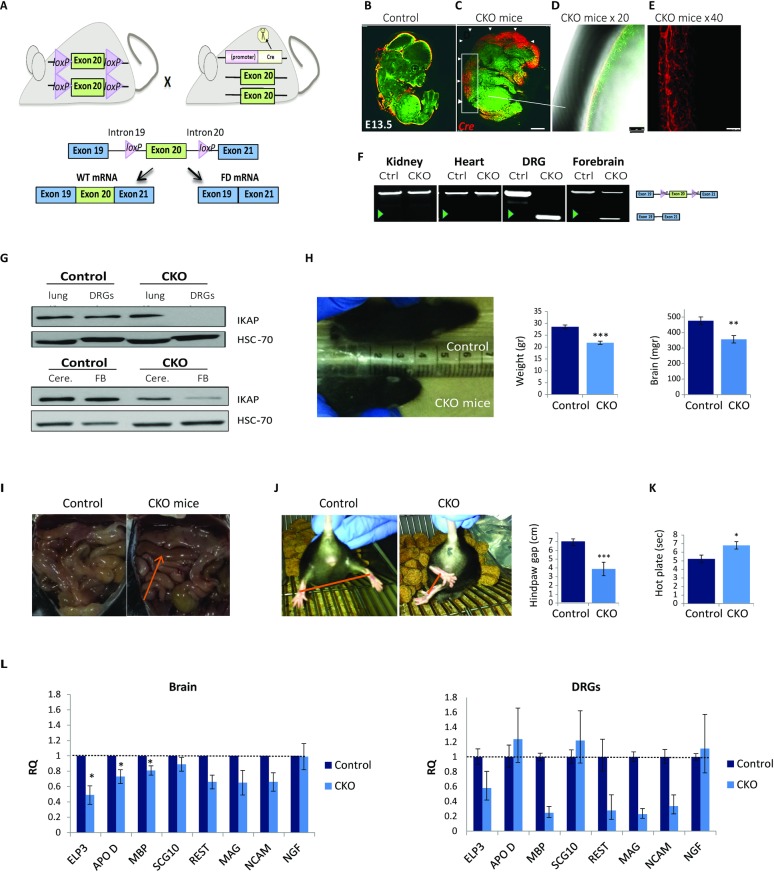
Generation of *Tyrp2-Cre;IKBKAP*^FDloxP/FDloxP^ (CKO^*Tyrp2*^ FD) mice. (**A**) Two *loxP* sequences were inserted in the introns flanking exon 20 of the *IKBKAP* gene (*IKBAKP*^FDloxP/FDloxP^ mouse, the mouse shown on the left). *IKBAKP*^FDloxP/FDloxP^ mice were mated with *Tyrp2-Cre* mice (the mouse on the right). The lower panel shows a schematic representation of the *IKBKAP*^*FDloxP/FDloxP*^ construct. *Cre* activation leads to exon 20 deletion in targeted tissues. (**B-E**) Whole-mount immunostaining using Cre (red) and Tuj-1 (green) antibodies of (B) control and (C-E) CKO^*Tyrp2*^ FD mice; enlargements are shown in D and E. (**F**) DNA from the indicated organs was extracted and analyzed to examine exon 20 deletion. Green arrowhead indicates removal of *IKBKAP* exon 20. (**G**) Western blot of IKAP in the lungs, DRGs, cerebellums (Cere.), and forebrains (FB) of control and CKO^*Tyrp2*^ FD mice. (**H**) Left panel: Photographs of CKO^*Tyrp2*^ FD and control littermates 10 days after birth (P10). Middle panel: Weights of CKO^*Tyrp2*^ FD and control mice (n = 40 per group, ***p<0.001). Right panel: Weights of brains from CKO^*Tyrp2*^ FD and control littermates (n = 10 per group, **p<0.01). (**I**) CKO^*Tyrp2*^ FD mice have brownish and swollen intestines (indicated by arrow). (**J**) Tail hanging test of three-month old CKO^*Tyrp2*^ FD and control littermates and plots of hindpaw gaps during tail hanging (n = 5 per group, ***p<0.001). (**K**) Hot-plate analgesia evaluation of thermal sensation and peripheral sensory nerve function of CKO^*Tyrp2*^ FD and control mice (n = 20 per group, *p<0.05). (**L**) qRT-PCR analysis of genes, known to be abnormally regulated in FD patients in brains (n = 3 of each group) and DRGs (pool of four mice for each group) of CKO^*Tyrp2*^ FD and control mice. Error bars represent ± SEM and for DRGs error bars represent technical standard deviation of three repeats.

Here we evaluated how IKAP mediates neurodegeneration in FD *in vivo* using a conditional knockout (CKO) mouse model in which exon 20 of *IKBKAP* is conditionally deleted in the brain and DRGs. *Tyrp2-Cre* [45] mice were mated with *IKBKAP*^FD*loxP*/FD*loxP*^ mice in which *IKBKAP* exon 20 flanked by *loxP* sites (Fig 1). In the resulting offspring (*Tyrp2-Cre*;*IKBKAP*^FD*loxP*/FD*loxP*^ termed the CKO^*Tyrp2*^ FD mice) *IKBKAP* exon 20 is deleted at an early differentiation stage of DRGs in the neural crest [45,46], a step at which IKAP expression was previously shown to be essential [43]. This deletion of IKAP was sufficient to generate the main FD symptoms in these mice including developmental delay, gastrointestinal dysfunction, motor discoordination problems, and reduced thermal perception. Importantly, these CKO^*Tyrp2*^ mice are viable and therefore enabled us to investigate the roles of IKAP in postmitotic neurons during postnatal stages. In CKO^*Tyrp2*^ FD mice, DRGs are grossly reduced in size relative to DRGs in control mice and overall the neuronal network formation is compromised. Our analysis of mutant DRGs revealed that IKAP deficiency resulted in less effective NGF axonal transport and suggests that IKAP is required for microtubule stabilization through effects on levels of HDAC6 and acetylated α-tubulin.

We further evaluated the ability of a potential therapy to alter neuronal maintenance. Previous FD drug discoveries have mainly attempted to elevate IKAP levels [47–50]. Phosphatidylserine (PS), a food supplement with no reported side effects, elevates *IKBKAP* transcription in cells generated from FD patients [10,51], in a mouse model for FD [52], and in preliminary results of clinical trials in FD patients [53]. Mechanistically, PS releases cells generated from FD patients from cell cycle arrest [10]. Also, treatment with PS upregulates *IKBKAP* transcription by activation of the MAPK/ERK pathway, which activates the transcription factors CREB and ELK1 that bind to the *IKBKAP* promoter region. This in turn enhances cell mobility [51]. Chronic administration of PS to normal adult rats promotes cell survival as shown by a significant increase in BrdU-positive proliferating cells [54], a reduction in pro-inflammatory signals [55], and inactivation of JNK and p38 signals after lipopolysaccharide treatment [56]. Here, using cells cultured from CKO^*Tyrp2*^ FD and wild-type mice, we examined how PS treatment influences neuronal maintenance. We observed that PS treatment stabilized microtubules by downregulating HDAC6 levels, elevating acetylated α-tubulin levels, and improving NGF axonal transport of DRG neurons. Therefore, PS treatment will likely enhance neuronal survival in FD patients and has potential for treatment of patients with other neurodegenerative disorders that share similar molecular pathways.

## Results

### Generation and characterization of a conditional knockout FD mouse

Total IKAP knockout in mice results in embryonic lethality between E10 to E12 (S1A Fig) [42,57]. In FD patients, the mutation in the *IKBKAP* gene primarily affects the nervous system [9] resulting in the abnormal fetal development and impaired postnatal maintenance of DRG neurons (S1A Fig) [13,18]. Thus, our goal was to establish a viable FD mouse model in which exon 20 is removed from the *IKBKAP* gene in the nervous system, mainly in DRGs and to study the role of IKAP in postmitotic neurons. We employed the *Tyrp2-Cre* line, previously reported to be active in pigmented cells of the eye, embryonic forebrain, and DRG [45]. Cre activation occurs on day E12.5, after DRG differentiation [45,46]. We established the CKO^*Tyrp2*^ FD by mating the humanized *IKBKAP* knock-in mouse [52] with a *Tyrp2-Cre* expressing mouse (Fig 1A and S1B Fig). The CKO^*Tyrp2*^ FD offspring were viable with no significant difference in survival compared to control littermates. Allele inheritance was according to Mendelian ratios and was verified using gDNA PCR (S1B Fig). We analyzed the CKO^*Tyrp2*^ FD offspring for Cre expression and Cre-mediated recombination of *IKBKAP* exon 20 using whole-mount immunostaining with the anti-Cre antibody (Fig 1B–1E), verified deletion of exon 20 by PCR of the genomic DNA (Fig 1F), and analyzed IKAP protein levels by western blot (Fig 1G). Control and CKO^*Tyrp2*^ FD mice had similar levels of IKAP in most tissues, such as the lung (Fig 1G), but in CKO^*Tyrp2*^ FD mice the levels of IKAP were negligible in the DRG (96% reduction compared to controls) and were reduced by more than 50% in the cerebellum and forebrain (Fig 1G).

We evaluated the CKO^*Tyrp2*^ FD mice for symptoms characteristic of FD. The CKO^*Tyrp2*^ FD mice had notable developmental delays. CKO^*Tyrp2*^ FD newborns were significantly smaller than control littermates (Fig 1H, left panel). At 2 months of age, CKO^*Tyrp2*^ FD mice had 20% lower body weight than control siblings (Fig 1H, middle panel; mean ± SEM, 21.8±1.28 g vs. 28.5±0.7 g, respectively, n = 40 per group, ***p<0.001) and CKO^*Tyrp2*^ FD brains weighed 25% less than brains of controls (Fig 1H, right panel; mean ± SEM, 0.37±0.02 g vs. 0.47±0.02 g, respectively, n = 10 per group, **p<0.01). These lower weights might result from problems in nursing and/or digestion, both characteristics of FD patients. Moreover, the CKO^*Tyrp2*^ FD mice had brownish and enlarged, swollen intestines (Fig 1I), which implies gastrointestinal dysmotility [58] as seen in FD patients [6].

Tail hanging evaluations revealed that 3-month-old CKO^*Tyrp2*^ FD mice show limb-clasping and abnormal posturing behavior when suspended by the tail, whereas we did not observe this in age-matched littermates (Fig 1J). In CKO^*Tyrp2*^ FD mice, the gap between two hindpaws when the mice were lifted was significantly shorter than that of control littermates (Fig 1J, n = 5, ***p<0.001). These behaviors are indicative of motor discoordination problems in CKO^*Tyrp2*^ FD mice [59]. Since FD patients exhibit inappropriate thermal and pain perception, we also performed hot plate analgesia assays as described previously [60]. At 3 months of age, CKO^*Tyrp2*^ FD mice exhibited less perception of sensation compared to age-matched controls (Fig 1K; mean ± SEM, 6.8±0.6 sec. vs. 5.2±0.4, n = 20 per group, *p<0.05).

We next examined whether the lack of IKAP alters expression levels of several genes known to be affected in FD patients and other FD mouse models [24,30,40,41,61]. RNA was extracted from the brains and DRGs of 3-month-old CKO^*Tyrp2*^ FD mice and control littermates. In the CKO^*Tyrp2*^ FD mouse brains and DRGs we observed lower levels of expression of FD-associated genes compared to controls (Fig 1L). The exception was *NGF*; levels were not significantly different between CKO^*Tyrp2*^ FD mice and controls (Fig 1L) Taken together, these results demonstrate that the CKO^*Tyrp2*^ FD mice have reduced IKAP levels in the brain and DRGs and exhibit many of the symptoms of FD.

### The role of *IKBKAP* in development and survival of neurons

**Fig 2:**
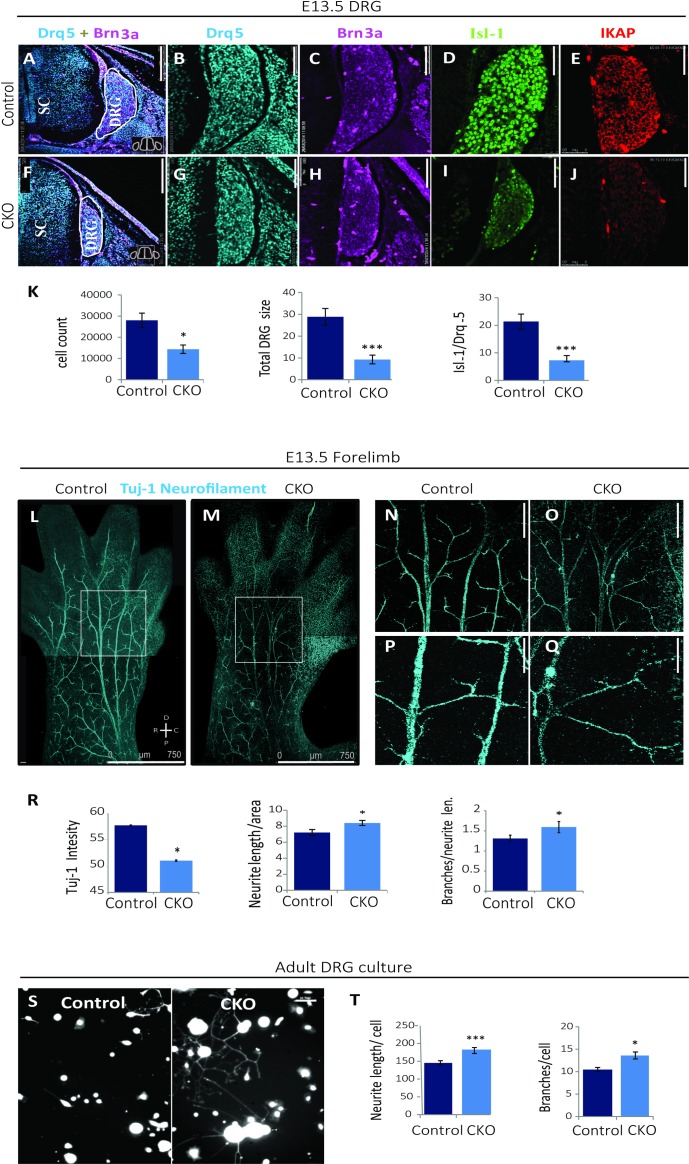
Deletion of *IKBKAP* exon 20 results in grossly reduced DRG size and interruption of peripheral projections. (**A-J**) Frozen cryo cross-sections of E13.5 CKO^*Tyrp2*^ FD and control littermate embryos were immunostained for DNA marker Drq5 (blue), DRG markers Brn3a (pink) and Isl-1 (green), and IKAP (red). Lumbar DRG cross-sections show decreases in DRG size as indicated by the gross morphology and decreased numbers of cells expressing Brn3a and Isl-1 in CKO^*Tyrp2*^ FD mice compared to controls. Scale bars 250 μm for panels A and F and 50 μm for panels B-E and G-J. (**K**) Quantification of average cell counts based on Drq5 immunostaining (n = 5 per group,*p<0.05), of total DRG size (in cm^3^, ***p<0.001), and of the Isl-1/Drq5 ratio for the CKO^*Tyrp2*^ FD DRGs compared to controls at E13.5 (***p<0.001). (**L-Q**) Embryos at E13.5 were whole-mount stained for neuronal marker Tuj-1 (blue). Scale bars: panels L and M, 750 μm; panels N and O, 250 μm; panels P and Q, 100 μm. (**R**) Quantification of Tuj-1 intensity (mean ROI intensity ± SEM,), average neurite length (mean ROI length/forelimb ROI size ± SEM), and numbers of branches (mean of total branches/neurite length ± SEM) based on ImageJ analysis of whole-mount staining (n = 40 per group, *p<0.05). (**S**) DRGs from 3 month-old CKO^*Tyrp2*^ FD and control mice were cultured for 24 h and stained using calcein to detect neurites and Hoechst dye to detect cell bodies. (**T**) Plots of neurite lengths and total numbers of branches normalized to cells number of DRGs cultured from 3 month-old CKO^*Tyrp2*^ FD and control mice (n = 300 per group, ***p<0.001 and *p<0.05, respectively). Error bars represent ± SEM.

The FD mutation leads to reduced DRG size and number [13,18–20,62]. We first utilized CKO^*Tyrp2*^ FD mice to analyze how the loss of *IKBKAP* disrupts DRG development and survival. IKAP was detected by immunohistochemistry and mouse anti-Isl-1 and anti-Brn3A antibodies were used as DRG markers (Fig 2A–2J). All analyses were performed at E13.5, a day after Cre activation [45,46]. In accordance with previous studies [43,44], IKAP was expressed in the DRG at E13.5 in control mice (Fig 2E); in contrast, very little IKAP was detected in the DRGs of CKO^*Tyrp2*^ FD mice at E13.5 (Fig 2J). DRGs were smaller in the CKO^*Tyrp2*^ FD mice compared to controls (Fig 2A–2J), and quantification of the number of cells in the DRG revealed massive cell loss during the embryonic development in the CKO^*Tyrp2*^ FD mice (Fig 2K). There was a 45% reduction in the average number of cells in the DRGs (*p<0.05), a 67% reduction in DRG area (in μm^3^, ***p<0.001), and a 63% reduction in the Isl-1/Drq5 ratio in CKO^*Tyrp2*^ FD mice compared to control littermates (***p<0.001). To determine whether IKAP depletion affects particular subpopulation of nociceptors and thermoreceptors neurons in the DRGs, we performed immunostaining using TrkA antibody. Our analysis did not reveal a difference in numbers of TrkA^+^ neurons in the CKO^*Tyrp2*^ FD embryos compared to control DRGs (S2 Fig).

We further evaluated DRG degeneration in the CKO^*Tyrp2*^ FD mice by analyzing DRG axonal projections in CKO^*Tyrp2*^ FD and control mice. Whole-mount neurofilament immunostaining was performed using an antibody to Tuj-1 in forelimbs of E13.5 control and CKO^*Tyrp2*^ FD mice to reveal the peripheral neuronal network innervation pattern. Tuj-1 staining revealed that nearly all peripheral projections were thinner in mutant forelimbs than in controls (Fig 2L–2Q) and that axon patterns were abnormal in CKO^*Tyrp2*^ FD mice. The Tuj-1 staining was 11% less intense in CKO^*Tyrp2*^ FD mice compared to that in control littermates (Fig 2R; mean ROI intensity ± SEM, 51.03±2.73 vs. 57.7±2.3; n = 5 per group, *p<0.05). These data are in agreement with analyses of induced pluripotent stem cells from FD patients, which revealed defects in the neurogenic propensity to form Tuj-1 sensory neurons [47]. We evaluated axonal projection guidance by measuring the average neurite length and by counting the numbers of branches. On average, neurites were 15% longer in CKO^*Tyrp2*^ FD forelimbs than in controls (Fig 2R; mean neurite length normalized to forelimb size ± SEM, 8.4±0.38 vs. 7.2±0.31 nm^3^; *p<0.05), and there were 21% more branches in CKO^*Tyrp2*^ FD neurites than in controls (Fig 2R; mean of total branches/neurite length ± SEM, 1.59±0.14 vs. 1.31±0.08 μm^3^; *p<0.05).

Proper functioning of the nervous system depends on the intricate array of connections that are formed during development [63]. Data from previous studies suggest that IKAP regulates peripheral nerve regeneration and that abnormal innervation may underlie the FD neurodegeneration [19,20]. To examine this hypothesis, we analyzed IKAP distribution in DRG cultures generated from the CKO^*Tyrp2*^ FD and control mice. The DRGs cultured from the CKO^*Tyrp2*^ FD mice had significantly more neurite outgrowth than did control DRGs (Fig 2S), suggesting that the lack of IKAP results in a stress response similar to a conditioning lesion [64] and/or alterations in axonal transport process [65]. Analysis of calcein staining revealed that neurite processes were longer (Fig 2T; mean neurite length ± SEM, 182.7±8.1 vs. 145±6.2 μm, ***p<0.001) and had excessive branching (Fig 2T; mean ± SEM of number of branches per cell, 13.7±0.76 vs. 10.4±0.48; *p<0.05) in CKO^*Tyrp2*^ FD DRGs compared to control cultures. These findings and the Tuj-1 immunostaining results indicate that downregulation of IKAP increases neurite outgrowth and neuronal branching.

**Fig 3:**
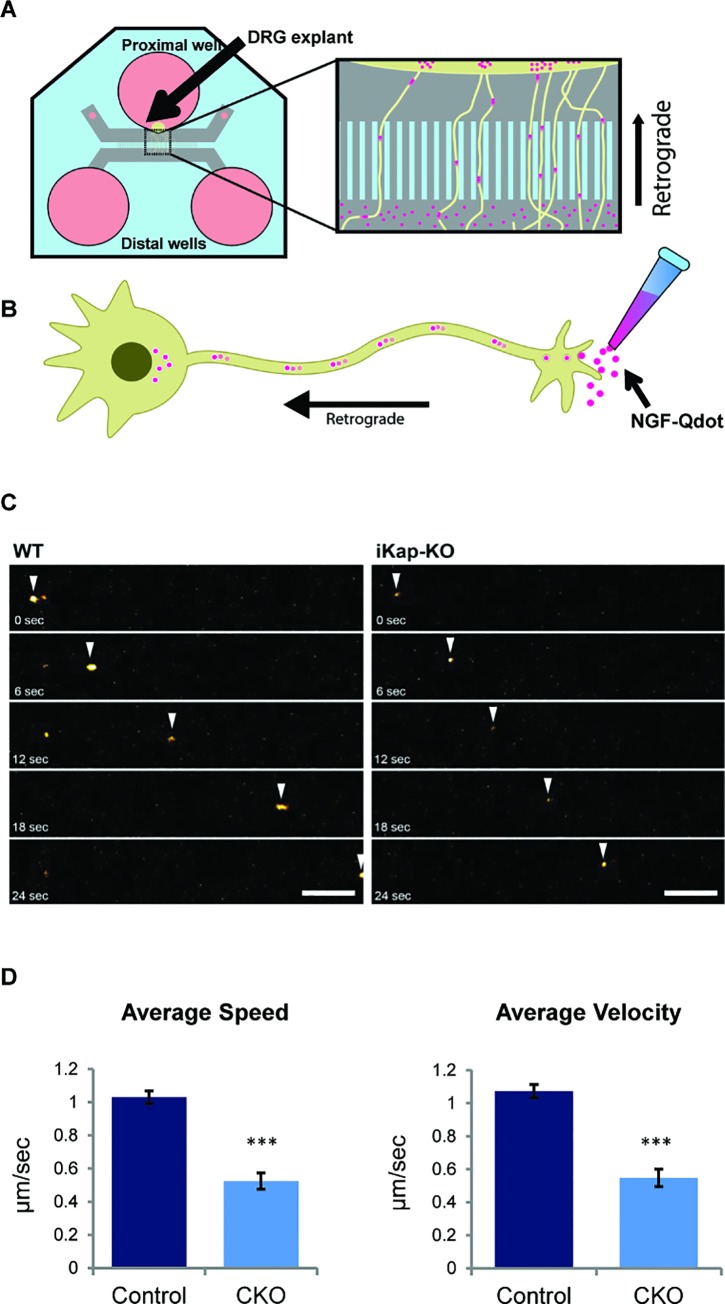
Deletion of *IKBKAP* exon 20 results in disrupted NGF axonal transport. (**A**) Schematic for the microfluidic system to track retrograde transport in DRG neurons. (**B**) Representation of retrograde axonal transport after addition of NGF-Qdot (pink). (**C**) DRG E13.5 explants were grown in microfluidic chambers, labeled NGF was added to the distal side, and bright field and fluorescent images were taken 24 h after plating. Arrows indicate transported particles. Scale bars: horizontal, 10 μm; vertical, 50 s. (**D**) The average velocities and speeds of labeled NGF were lower in CKO^*Tyrp2*^ FD DRGs than control DRGs (***p<0.001). Error bars represent ± SEM.

Neurotrophic factors such as nerve growth factor (NGF) and adhesion molecules like NCAM can regulate axon growth. Interestingly, NCAM and NGF expression levels are correlated with IKAP levels [19,24,32,44]. The levels of *NCAM* mRNA were notably lower in the extracts of DRGs from CKO^*Tyrp2*^ FD compared to control mice; however, *NGF* levels were not significantly different (Fig 1L). We speculated though that subcellular localization of NGF might be altered in CKO^*Tyrp2*^ FD mice, as neuronal survival, growth, and wiring depend on NGF localization and transport along the axon [66,67]. To examine how lack of IKAP affects NGF trafficking along the DRGs, DRG E13.5 explants were grown in microfluidic chambers. Labeled NGF was added to the distal side of the chamber, and live cell imaging was used to track the retrograde transport of NGF signaling endosomes as we did previously [68,69] (Fig 3A–3C). Analyses revealed 50% decreases in the average instantaneous velocity (mean ± SEM, 0.52±0.05 vs. 1.03±0.04 μm/sec, respectively, ***p<0.001) and in the average speed (0.05±0.05 vs. 1.03±0.04 μm/sec, respectively, ***p<0.001) of NGF transport in CKO^*Tyrp2*^ FD compared to control DRGs (Fig 3D). Thus, CKO^*Tyrp2*^ FD DRGs have fewer signaling endosomes per axon and impaired NGF transport compared to DRGs cultured from control littermates.

### IKAP deficiency results in microtubule destabilization

**Fig 4:**
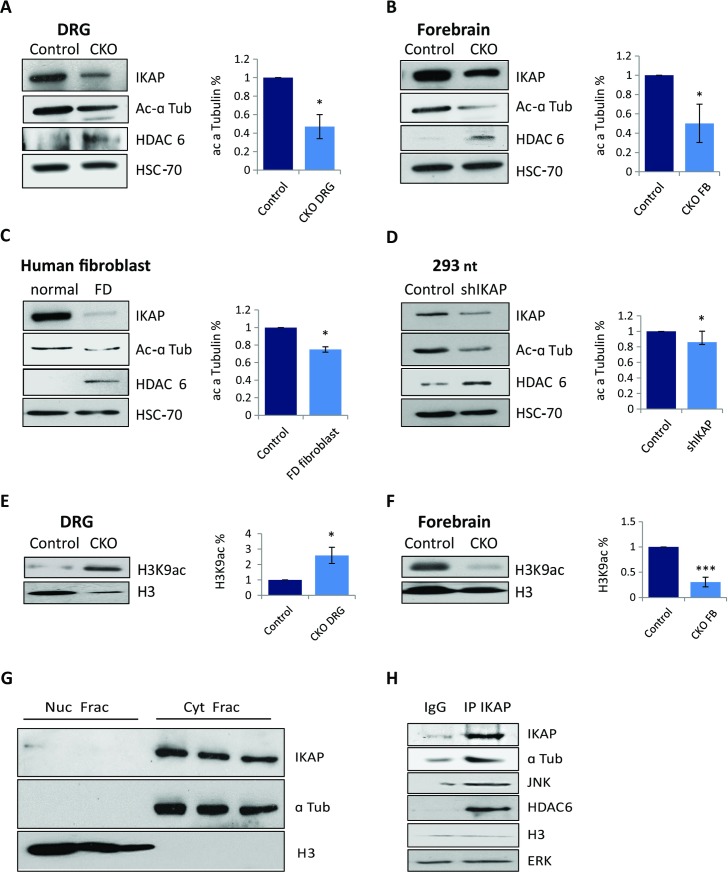
IKAP is a cytoplasmic protein that interacts with α-tubulin and HDAC6. (**A-D**) Right panels: Representative western blots for IKAP, acetylated α-tubulin, and HDAC6. Left panels: Relative levels of acetylated α-tubulin in control (set to 1) and IKAP-deficient samples from (**A**) control and CKO^*Tyrp2*^ FD DRG extracts, (**B**) control and CKO^*Tyrp2*^ FD forebrains, (**C**) extracts generated from fibroblasts from normal controls and an FD patient, and (**D**) HEK 293nt controls and shIKAP cells. HSC-70 levels were used as a protein loading control. Quantifications are of three biological replicates (*p<0.05). (**E-F**) Western blots and quantification of histone H3K9ac levels in (**E**) DRGs (*p<0.05) and (**F**) forebrain CKO^*Tyrp2*^ FD DRGs and control DRGs (***p<0.001). (**G**) Western blots of nuclear (Nuc) and cytoplasmic (Cyt) fractions of HEK 293nt lysates. Histone H3 was present only in the nuclear fraction and α-tubulin only in the cytoplasmic fraction. (**H**) HEK 293nt cell lysate was immunoprecipitated with anti-IKAP antibody followed by immunoblot analysis for indicated proteins. Error bars represent SEM.

Alterations in the axonal transport process are accompanied by a decrease in acetylation of α-tubulin in many neurodegenerative diseases [66,70–72]. Acetylated α-tubulin and intracellular transport are vital to neuronal function and survival [67,73] and for microtubule stabilization [74,75]. Microtubules form the backbone of axons and are configured by the polymerization of α- and β-tubulin dimers. Previous studies have reported contradictory findings on acetylation of α-tubulin in FD [20,23,30,37,38,40,44,76]. To evaluate whether the axonal transport defects we detected in CKO^*Tyrp2*^ FD DRGs are due to the effect of IKAP on acetylation of α-tubulin, we used immunoblot assays to quantify acetylated α-tubulin in FD CKO^*Tyrp2*^ mouse DRGs and brains, in fibroblasts derived from FD patients, and in HEK 293nt cells in which IKAP levels were reduced by stable expression of a shRNA targeted to the *IKBKAP* mRNA (shIKAP) (Fig 4A–4D; *p<0.05). We observed a 53% decrease in acetylated α-tubulin in DRGs of CKO^*Tyrp2*^ FD mice relative to controls (Fig 4A; *p<0.05), and a 49% decrease in the forebrain of CKO^*Tyrp2*^ FD mice relative to controls (Fig 4B; *p<0.05). In fibroblasts derived from FD patients compared to those from normal controls, there was a 25% reduction in acetylated α-tubulin (Fig 4C; *p<0.05). HEK 293nt cells deficient in IKAP had 14% less acetylated α-tubulin than control cells (Fig 4D; *p<0.05). Data from these experiments indicated that depletion of exon 20 of *IKBKAP* resulted in a more significant decreased in acetylated α-tubulin levels in neuronal tissues than in non-neuronal cells. These differences in the acetylated α-tubulin levels are likely due to the morphology of neurons, especially the DRGs, which are highly polarized cells with very long axons, made of tubulin subunits. The less dramatic differences in levels of acetylated α-tubulin in fibroblasts and HEK 293nt cells that are deficient in IKAP compared to controls illustrates why alterations in acetylated α-tubulin levels are not necessarily detected in FD patients and might explain the neuronal specificity of the FD phenotype.

Reductions in acetylated α-tubulin were previously reported to be accompanied by increased HDAC6 expression [77]. HDAC6 is the major α-tubulin deacetylase, and its expression is abnormally high in patients with neurodegenerative diseases, including Parkinson’s, amyotrophic lateral sclerosis, and Alzheimer’s, and with cancer and pathological autoimmune responses [78]. In each of our models of IKAP deficiency, we observed increased HDAC6 levels relative to controls (Fig 4A–4D).

IKAP was previously suggested to impact histone acetylation as part of the Elongator complex [26–29], and levels of mRNA encoding the catalytic histone acetyltransferase subunit of Elongator protein 3 (ELP3) were significantly downregulated in CKO^*Tyrp2*^ FD mice compared to control littermates (Fig 1L). We therefore examined the effect of IKAP depletion on acetylation of histone H3K9. Interestingly, we observed higher H3K9ac levels in CKO^*Tyrp2*^ FD DRGs than in control DRGs (Fig 4E; *p<0.05), and the opposite in neurons from the forebrain (Fig 4F; ***p<0.001). These results are in line with the previous finding that H3K9 acetylation is elevated in DRGs but not in the central nervous system during neuronal damage [79,80]. Thus, IKAP has indirect and tissue-specific functions in neuronal development and function.

### IKAP interacts with acetylated α-tubulin

To further explore the nature of the link between IKAP, microtubules, and histones, we examined fractionated nuclear and cytoplasmic lysates and performed co-immunoprecipitation assay for endogenous IKAP. Since the IKAP effect on acetylated α-tubulin was similar in different cell types (Fig 4A–4D), these experiments were performed in HEK 293nt cells. α-tubulin was used as a marker for cytoplasmic lysate fraction and histone H3 as a nuclear marker. In these extracts, no cross-contamination of fractions was observed, and IKAP was restricted to the cytoplasmic fraction (Fig 4G). DNA was fragmented by limited sonication as described [81], and anti-IKAP antibody was used to precipitate endogenous IKAP. We observed interactions between IKAP and α-tubulin, JNK, and HDAC6 but no enrichment for histone H3 or ERK (Fig 4H). A reciprocal immunoprecipitation assay using anti-HDAC6 also precipitated α-tubulin and IKAP (S3 Fig). Thus, IKAP is found in a cytoplasmic protein complex together with α-tubulin, JNK, and HDAC6.

### Phosphatidylserine ameliorates symptoms of molecular neurodegeneration of CKO^*Tyrp2*^ FD mice

PS is a phospholipid that is normally located on the cytoplasmic face of the lipid layer. It is exposed on the outer leaflet of the plasma membrane during apoptosis where it recognized by PS receptors on scavenger macrophages [82]. Though its protective mechanism is not understood, PS administration promotes cell survival [54–56] and was found to elevate IKAP levels in cell line derived from FD patients [10,51], in a mouse model for FD [52] and in preliminary results of clinical trials [53]. Protection may reflect the ability of PS to stimulate IKAP transcription by activating the MAPK/ERK pathway or conceivably exogenous PS may relieve apoptotic stress on neurons and other cell types by saturating macrophage PS receptors [51].

**Fig 5:**
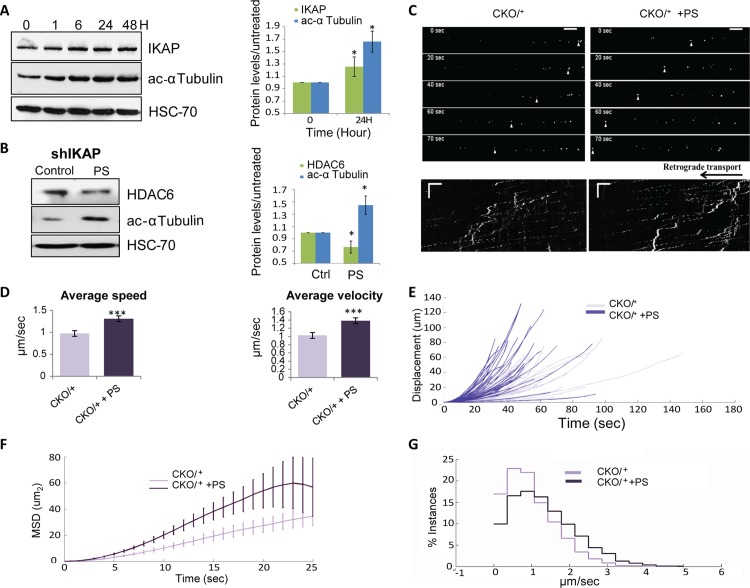
Phosphatidylserine elevates acetylated α-tubulin levels and rescues axonal transport. (**A**) HEK 293nt cells were treated with 200 μg/μl PS for the indicated time. Left panel: Proteins were extracted, and IKAP, acetylated α-tubulin, and HSC-70 levels were analyzed by western blot. Right panel: Quantification of 24-h data with control levels set to 1 (n = 3, *p<0.05). (**B**) Western blot of extract of HEK 293nt shIKAP cells treated with 200 μg/μl PS for 24 h. (**C-G**) PS alters NGF transport in DRG explants culture from CKO/^+^ embryos. Labeled NGF was added to the distal side of the culture, and bright field and fluorescent images were taken 24 hours after addition of PS or vehicle. (C) NGF-Qdot transport was imaged in DRG neurons upon PS treatment. The arrowheads track representative faster Q-dots along the axon of PS treatment neurons. Below is a representative kymograph demonstrating faster NGF-Qdot transport in PS-treated compared to control cells. (**D**) Mean average velocities and speeds (***p<0.001), (**E**) displacement, and (**F**) mean square displacement plotted vs. time of labeled NGF in CKO/^+^ DRG cultures treated with PS or vehicle. Error bars represent SEM. (**G**) Comparisons of the distribution profiles for instantaneous velocities show that PS treatment induces an overall shift toward faster transport velocities.

We assume that the beneficial effects of PS result from an increase in IKAP levels in treated cells. Therefore, we first evaluated the effect of PS on levels of acetylated α–tubulin in HEK 293nt cells. Treatment of cells with 200 μg/ml PS elevated acetylated α-tubulin levels by 50% after 24 h (Fig 5A; *p<0.05). We then examined the impact of PS administration on HEK 293nt cells that stably express the shIKAP. In these cells depleted of IKAP, PS treatment resulted in a 44% increase in acetylated α-tubulin levels (Fig 5B). PS also significantly downregulated HDAC6 levels in the shIKAP cells compared to vehicle only treated cells (Fig 5B). This indicates that PS can acts as HDAC6 inhibitor.

We next evaluated PS treatment on fibroblasts extracted from FD patients. An increase in acetylated α-tubulin and IKAP levels were also observed as a result of PS administration (S4A Fig). Trichostatin A (TSA), a general HDAC inhibitor [83,84] was used as a positive control and had an effect similar to that of treatment with PS on IKAP and acetylated α-tubulin levels (S4A Fig). As another approach to characterize the role of IKAP and the ability of PS to stabilize tubulin, we performed a wound-healing assay. Wound healing is central in a variety of pathologic and physiologic processes including cell growth, differentiation, and cell-cell communication and is a process typically associated with the ability of cells to project tubulin branches. IKAP-depleted cells were previously shown to have impaired migration in wound healing assays [32,37]. The shIKAP 293nt HEK cells were grown to confluence in complete media with or without PS, a wound was made using 96-well Wound Maker, and cells were imaged as described [51]. Images and measurements of the wound size as a function of time indicated that in cells that are IKAP deficient migration was enhanced by treatment with either PS or TSA (S4B Fig). These findings suggest that PS works in a similar manner to an HDAC inhibitors and improves the FD phenotype.

We next analyzed the ability of PS treatment to improve axonal transport of the CKO^*Tyrp2*^ FD mouse. No significant effects were detected on FD symptoms (S5 Fig). In this model, exon 20 of the *IKBKAP* gene is conditionally deleted. This means that PS treatment cannot increase levels of full-length IKAP in these mice. In contrast, in heterozygotes (CKO/^+^; in which only one of the *IKBKAP* alleles has a conditionally deleted exon 20) and in wild-type mice, it is possible for PS treatment to elevate IKAP levels from the normal allele. We therefore examined the effect of PS administration on axonal transport in DRGs taken from wild-type (S6A–S6E Fig) and CKO/^+^ mice (Fig 5C–5G). DRG explants were grown in microfluidic chambers and labeled NGF was added to the distal side. Analysis of the retrograde transport of NGF signaling endosomes along the axon, 24 hours after PS addition indicated enhancement of trafficking compared to vehicle-treated controls both in CKO/^+^ explants (Fig 5C–5G; ***p<0.001) and wild-type explants (S6A–S6E Fig). The average instantaneous velocity of NGF transport after PS administration was significantly higher than in vehicle only treated CKO/^+^ controls (mean ± SEM, 1.4±0.07 vs. 1.03±0.07 μm/sec, respectively, ***p<0.001) as was the average speed (1.31±0.06 vs. 0.97±0.06, μm/sec, respectively, ***p<0.001). Moreover, the mean squared displacements and displacement as a function of time indicated that PS treatment resulted in more rapid transport of NGF across the axons (Fig 5E and 5F and S6C and S6D Fig). No significant differences were found between CKO/+ and normal mice (S7 Fig). These experiments show that PS increases levels of IKAP in mice with at least one normal *IKBKAP* allele, resulting in amelioration of damaged NGF axonal transport and an increase in transport efficiency.

**Fig 6:**
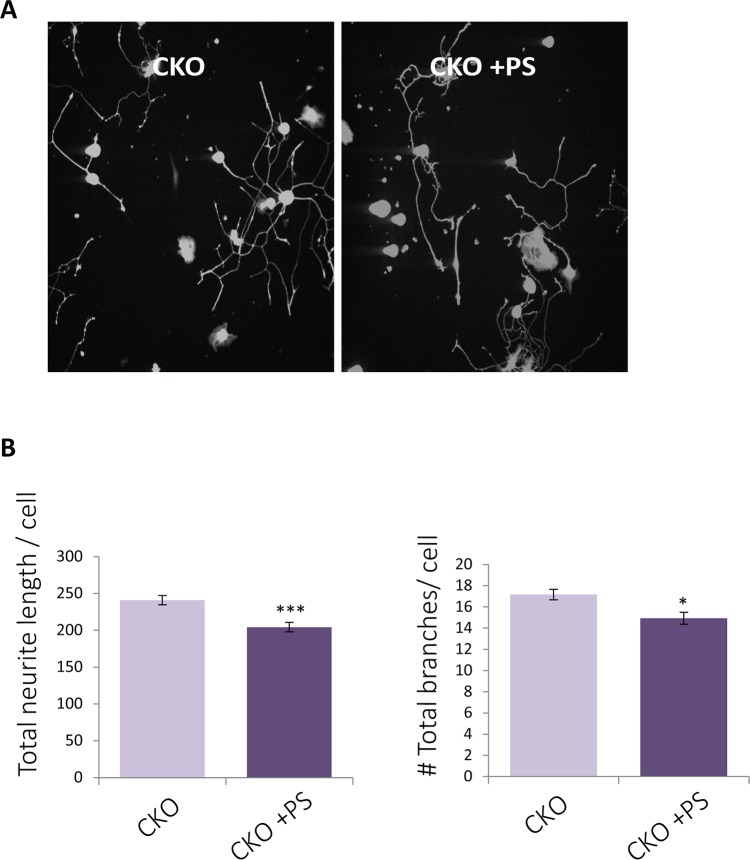
Phosphatidylserine improves neuritis outgrowth. (**A**) DRGs were extracted from 3 month-old CKO^*Tyrp2*^ FD mice and grown in culture. Cells were treated with 200 μg/ul PS for 24 hrs. Cultures were stained with calcein to detect neurites and Hoechst dye to detect cell bodies. (**B**). Neurite length (***p<0.001) and total branches (*p<0.05) were compared to untreated and vehicle only controls and normalized to number of DRGs. Error bars represent ± SEM.

Finally, we examined the ability of PS to improve DRG culture outgrowth. DRGs cultured from the CKO^*Tyrp2*^ FD mice and treated with PS had significantly less neurite outgrowth than did vehicle only controls (Fig 6A and 6B). Analysis of calcein staining revealed that neurite processes were shorter in CKO^*Tyrp2*^ FD DRGs treated with PS (Fig 6B; mean neurite length ± SEM, 204.3±6.3 vs. 240.9.±6.5 μm, ***p<0.001) and had less branching per cell (Fig 6B; mean ± SEM 14.9±0.5 vs. 17.1±0.5; **p<0.01) compared to vehicle-treated control cultures. As PS cannot increase IKAP levels in these mice, its affect is most likely independent of IKAP and may involve HDAC6 inhibition and microtubule stability. Indeed, PS had an affect on α-tubulin dynamics in nocodazole-treated fibroblasts derived from FD patients compare to normal controls. Nocodazole treatment collapses the microtubule network as reveled by α-tubulin staining (S8 Fig). Cells were treated with 1.0 μM nocodazole and either 200 μg/ml PS or vehicle. Fibroblasts derived from FD patients and from normal controls showed greater resistance to nocodazole treatment and less microtubule depolymerisation when the nocodazole treatment was combined with PS treatment compared to cells treated with nocodazole and vehicle (S8 Fig).

## Discussion

IKAP is a well-studied protein, yet its role in neuronal survival remains controversial. Although it has been known for decades that a mutation that results in mis-splicing of the gene that encodes IKAP causes FD, the molecular mechanism that leads to the progressive neurodegeneration observed in FD patients has been unclear. There have been previous attempts to generate a mouse model for FD. Knock-in mice in which the human *IKBKAP* locus with the mutation observed in FD patients replaces the reciprocal mouse genomic sequences are fully viable and do not show any FD symptoms other than splicing of *IKBKAP* in a tissue-specific manner [10,52,85]. *IKBKAP* total knockout results in embryonic lethality between E10 and E12 [42,57]. The conditional knockout of *IKBKAP* using a *Cre* expression system under control of the *Wnt-1* promoter resulted in mice that died a few days after birth [20,44]. Recently a combined heterozygote model in which one *IKBKAP* allele is knocked-in with the FD mutation and the second is knocked-out resulted in a mouse that recapitulates many phenotypic features of FD and recreates the same tissue-specific mis-splicing defect seen in FD patients [62]. As IKAP depletion occurs early in embryogenesis in these mice, the roles of IKAP in DRG development and early differentiation cannot be separated from its role in neurodegeneration. Thus, in order to bypass the *IKBKAP*^*-/-*^ and *Wnt-1* lethality and study the effect of IKAP on neurodegeneration, we established a conditional knockout transgenic mouse line using *Tyrp-2* promoter. This conditional knockout results in loss of IKAP at E12.5, after DRG maturation. These mice are viable and mimic the human FD phenotype. These CKO^*Tyrp2*^ FD mice enabled us to study the role of IKAP in neuronal maintenance *in vivo* and to establish the mechanism by which PS improves symptoms of neurodegeneration.

We first assessed the function of IKAP in DRG development and survival *in vivo*. First, our data indicate that depletion of IKAP in the brain results in DRGs with grossly reduced size, aberrant peripheral innervations, and excessive axonal growth when compared to controls. Second, DRGs from CKO^*Tyrp2*^ FD mice exhibit longer neurites and more branches than DRGs derived from normal littermates. These findings are similar to those reported based on analyses of chick and mouse models of FD that showed that IKAP is crucial for peripheral neuron target innervations and NGF signaling [19,20,42,43]. Third, DRG from CKO^*Tyrp2*^ FD mice had less acetylated α-tubulin than control littermates and impaired NGF axonal transport. Reductions in the acetylated form of α-tubulin were also observed in SGs from these mice. Our data are in agreement with a previous report by Gardiner et al. that demonstrated that retrograde transport was impaired in FD patients [23]. That IKAP is involved in axonal transport was suggested by the finding that exogenously expressed IKAP C-terminal domain co-purifies with the heavy chain of the motor protein dynein [32] and that the absence of IKAP disrupts co-localization of dynein with phosphorylated JNK along axons [19] and causes alterations in expression of several genes important in axonal transport [24].

DRGs are highly polarized cells with very long axons. The structural integrity of these axons is dependent on efficient intracellular transport of cargoes such as neurotrophic factors manufactured in their distant targets cells. Thus, DRGs may be more vulnerable to alterations in axonal transport process than are other neuronal cells types. In DRGs cultured from IKAP-deficient CKO^*Tyrp2*^ FD mice, NGF transport was impaired relative to control DRGs that express normal levels of IKAP. The unstable neuronal networks formed in IKAP-deficient conditions may underlie the massive DRG neurodegeneration progression observed in FD patients.

There is a well-established link between α-tubulin acetylation, protein trafficking, microtubule stability, and neurodegeneration [70–72,74,75]. Reduced acetylation of α-tubulin correlates with axonal transport defects in Alzheimer’s and Huntington’s disease patients [66] and treatment with an HDAC inhibitor was shown to rescue vesicular transport in a Huntington’s disease model [86]. Moreover, in several FD studies abnormally low levels of α-tubulin acetylation have been reported [38,76]. In DRGs and forebrain neurons from our CKO^*Tyrp2*^ FD mice, we observed significantly increased HDAC6 levels and significantly decreased acetylated α-tubulin levels compared to control littermates. How IKAP levels affect HDAC6 expression remains unclear, although we demonstrated that endogenous IKAP co-purified with HDAC6 and α-tubulin, suggesting that a protein-protein complex might ensure that levels are balanced. Another possibility is that the decrease in acetylated α-tubulin itself stimulates the neuroprotective role of HDAC6, as a previous study revealed a robust and selective increase in HDAC6 expression following neuronal injury [87]. Thus, the co-purification observed might be due to binding of IKAP and HDAC6 to the same substrate. Furthermore, HDAC6 elevation may underlie the excessive axonal growth since HDAC6 overexpression was shown to increase neurite length [88].

Although IKAP has been suggested to serve as a scaffold protein of the Elongator complex that is responsible for chromatin remodeling during transcription [26,37,89], in other studies IKAP has been shown to be restricted to the cytoplasm [31]. One of the functions of the Elongator complex is acetylation of histone H3 [90], particularly at K9 [37,90]. Indeed, we observed that the levels of ELP3, a component of the Elongator complex, were significantly downregulated upon IKAP depletion. However, our findings suggest that the effect of IKAP on histone acetylation must be indirect. We did not detect IKAP in nuclear fractions, IKAP did not precipitate histone H3, and the impact on H3K9ac of IKAP depletion depended on the tissue. Mass spectrometry analysis of proteins that precipitated with the C-terminal domain of IKAP identified primarily cytoplasmic proteins [32], and exogenous IKAP precipitated and partly co-localized with α-tubulin [38]. We assume that changes in H3K9ac levels probably mediate chromatin remodeling during neuronal degeneration in FD; thus, IKAP has an indirect impact on histone H3K9 acetylation. Phosphorylated JNK was previously suggested to mediate the interaction between IKAP and α-tubulin [19,23,31], and in our experiments, JNK was precipitated with the anti-IKAP antibody.

**Fig 7:**
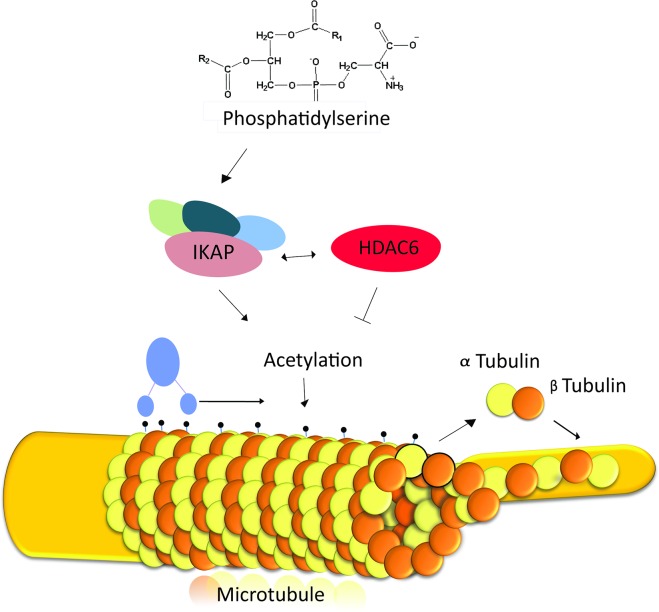
A suggested model for the effect of PS on impaired axonal transport in FD. Phosphatidylserine affects axonal transport and microtubule stabilization by balancing the interplay of IKAP and HDAC6 levels. IKAP is part of the Elongator complex that contains the catalytic acetyltransferase subunit of ELP3. The Elongator complex acetylates α-tubulin, which is crucial for dynein movement and polymerization of microtubules. HDAC6 destabilizes acetylated α-tubulin, and its levels are influenced by levels of IKAP and other Elongator components. Phosphatidylserine elevates IKAP levels and downregulates HDAC6 levels and thus facilitates axonal transport and microtubule stabilization.

Recently it was demonstrated that *IKBKAP* mRNA levels are downregulated in FD patients during crisis [91], which emphasizes that treatments that elevate transcription of *IKBKAP* should be an effective therapy for FD. PS was previously found to stimulate the *IKBKAP* transcription [51] and to elevate IKAP levels *in vitro* and *in vivo* [10,52]. Here we explored the effects of PS treatment on DRG explants from mice that have only one functional *IKBKAP* allele and wild-type mice and found that PS treatment enhances NGF retrograde axonal transport, and improves axonal outgrowth. We also identified PS downregulates HDAC6 levels and elevate acetylated α-tubulin levels. We assume that PS treatment increased levels of acetylated α-tubulin levels through its indirect inhibitory effects on HDAC6 as well as activation of Elongator complex and the ELP3 acetyltransferase activities (Fig 7). We showed that in several assays PS treatment is similar to treatment with the well-characterized HDAC inhibitor TSA.

Both PS and an HDAC inhibitor were demonstrated to benefit patients with depression and mood disorder [92] and enhance memory formation [93,94]. The responses to treatment with broad-spectrum HDAC inhibitors are heterogeneous, protective in some cases and detrimental in others [95]. Recently PS treatment has been reported to improve memory in Alzheimer’s patients and to reduce hippocampal inflammation and free radical production in a rat model of Alzheimer’s disease [96]. The fact that PS has been used clinically with no serious side effects and promotes neuron survival and axonal transport suggest that PS might be relevant as treatment for other neurodegenerative diseases that share similar pathologic and molecular mechanisms. Additional clinical studies are warranted.

## Materials and Methods

### Ethics statement

All animal work and all procedures followed guidelines according to the NRC—guide for care and use of laboratory animals. The study was approved by the Institutional Animal Care and Use Committee (IACUC) of Tel Aviv University with the approval numbers: M-12-047 (previous) and M-15-015 (current), and measures were taken to minimize pain and discomfort.

### Antibodies

Antibodies used for immunofluorescent staining were as follows: Brn3a (Chemicon, a gift from the Miguel Weil lab, Tel Aviv University), IKAP (Anaspec or Santa Cruz), DRQ5 (Cell Signaling), Isl-1 (DSHB) and TrkA (Almone). Antibodies used for whole-mount staining were anti-Cre (Abcam) and neuronal class III -tubulin (anti-Tuj-1; Covance) which were kind gifts from the Keren Avraham lab (Tel Aviv University) and the Avraham Yaron lab (Weizmann Institute). Secondary antibodies were donkey anti-rabbit conjugated to Alexa-594, donkey anti-mouse Alexa-488, and donkey anti-goat Alexa-647 (all from Invitrogen and used 1∶1000). DRG cultures were stained using Hoechst dye and calcein (Thermo Scientific).

For immunoblotting, the primary antibodies used were: anti-IKAP (Anaspec), anti-acetylated α-tubulin (Sigma), anti-HDAC6 (Abcam), anti-Hsc70 (Santa Cruz Biotechnology), anti-GAPDH (GenScript), anti-α-tubulin (Abcam), anti-histone H3K9 (Abcam), anti-histone H3 (Abcam), anti-histone H4 (Millipore), anti-JNK (Santa Cruz Biotechnology), anti-pJNK (Santa Cruz Biotechnology), anti-ERK (Cell Signaling), anti-pERK (Santa Cruz Biotechnology). Secondary antibodies donkey anti-rabbit IgG HRP (Abcam), donkey anti-goat IgG (Abcam), or goat anti-mouse IgG (Jackson) were used as appropriate.

### PS treatment

InCog, a lipid composition containing PS-omega 3, DHA-enriched, referred to here as PS, was dissolved in organic solvent medium chain triglycerides (MCT). Both PS and MCT were obtained from Enzymotec. In all treatments PS was used at 200 μg/ml and was compared to treatment with solvent as a control.

### Mouse lines

The mouse lines employed in this study, *Tyrp2-Cre[45]* and *IKBKAP*^FD*loxP*/FD*loxP*^[52], have been previously described. These lines were used to establish *Tyrp2-Cre*;*IKBKAP*^FD*loxP*/FD*loxP*^ somatic mutants. In all experiments *IKBKAP*^FD*loxP*/FD*loxP*^ littermates were used as controls. All animal work and all procedures were conducted according to national and international guidelines, and procedures were approved by the Tel Aviv University review board and measures were taken to minimize pain and discomfort.

### Genotyping and DNA purification

Genotypes were determined by PCR analysis of genomic DNA from tail slips using the High Pure PCR Template Preparation Kit (Roche) or KAPA mouse genotyping (Biosystems) according to the manufacturer’s instructions. To distinguish *IKBKAP* wild-type, heterozygous, and homozygous mice, we used primers 5’-GATAGCTAGTCTGTGTTGTAATG-3’ and 5’-CCCTCGTGTGCCCTCAGTG-3’. Wild-type and homozygous *IKBKAP*^FD*loxP*/FD*loxP*^ mice have genomic fragments of 1140 and 1392 bp, respectively, whereas heterozygous *IKBKAP*^FD*loxP*/+^ mice display both fragments. *Cre* was detected using 5’-CCGCAGAACCTGAAGATGTTC-3’ and 5’-TCATCAGCTACACCAGAGACG-3’. Heterozygous *Cre* mice display a 500-bp fragment.

### Behavioral and phenotype evaluations

CKO^*Tyrp2*^ FD and control mice were evaluated for their nociceptive threshold to radiant heat using the hot plate (Ugo Basile) paw withdrawal test, as previously described[60]. Briefly, a 40-cm high Plexiglas cylinder was suspended over the 55°C hot plate to give a latency of about 10 seconds for control mice. Withdrawal latency was defined as time between placement on the hot plate and time of withdrawal and licking of hindpaw. Each animal was tested twice, separated by a 30-min rest interval. Student's t-test was used to calculate significance of differences between CKO^*Tyrp2*^ FD mice and controls. The four-limb tail hanging hindpaw gap evaluations were conducted using Image J.

### RT-qPCR

RNA was extracted from CKO^*Tyrp2*^ FD and control mice using TRI reagent (Sigma) and reverse-transcribed using the SuperScript III First Strand kit (Invitrogen) with an oligo (dT) reverse primer. A qPCR analysis of mRNA expression from mouse brain or DRGs tissue samples were conducted using KAPA SYBR fast qPCR master mix (Kapa Biosystems) in a StepOne plus thermocycler PCR machine (Applied Biosystems) according to the manufacturer’s instructions. Expression levels of *PPIA* and *GAPDH* genes were used as endogenous controls. Brain samples were assayed in triplicate and DRGs were assayed in pulls from four mice. Primers are listed in the supplementary material (S1 Table).

### Immunostaining

Immunofluorescence analysis was performed on 10-μm paraffin or cryo frozen sections as previously described [97]. Stains were analyzed using Image J. For each genotype, we evaluated three to six mice. For each DRG, we counted four to six slices. All fluorescent images were obtained using Super-Resolution Microscope Leica TCS STED or Zeiss confocal microscopy LSM 510.

### Whole-mount staining

Whole-mount samples were prepared as follows: at E13.5 embryos were dissected and immediately fixed in 4% paraformaldehyde for 6 h 4°C. Embryos were washed and dehydrated through a series of methanol, Dent’s bleach (methanol:DMSO:hydrogen peroxide) and in a descending series of methanol concentrations. Embryos were blocked and stained with primary antibody followed by second antibodies overnight at 4°C. Embryos were washed in PBS and incubated in the diluted secondary antibody in blocking solution and then rinsed in a glycerol solution. Embryos were flattened on the basal side in Lab-Tek coverglass sealed mounted chambers (Nunc) for observation. For whole-mount statistical analysis, we counted eight to ten neuritis of five forelimbs stained for each group.

### DRG culture

Adult DRG cultures were cultured as described [98]. Neurons were dissociated by incubation with 100 U of papain followed by 1 mg/ml collagenase-II and 1.2 mg/ml dispase. Then DRGs were triturated in HBSS, 10 mM Glucose, and 5 mM HEPES (pH 7.35). Cells were recovered through percoll, plated on laminin, and grown in F12 medium for 48 hr. Neurite outgrowth assays were monitored on a Revolution XD automated imaging system (Andor) using calcein AM (C3100MP) and Hoechst dye. For rescue experiments, DRG neurons were treated with either PS or an equivalent amount of vehicle for 12 h in DRG medium without NGF to exclude outgrowth effects by NGF. The system automatically collected 10 images per well. Automated analyses of the results were performed using WIS-Neuromath software developed at the Weizmann Institute (www.weizmann.ac.il/vet/IC/software/wis-neuromath) [99]. For DRG culture statistical analysis, we filmed on average 70 fields of four different experiments for each group.

### Cell culture, shRNA transfection and RT-PCR

Human FD fibroblast cells were obtained from the appendices of FD patients and immortalized using telomerase activation. HEK 293nt cells and human FD and normal fibroblast cell lines were cultured in Dulbecco’s modified Eagle’s medium, supplemented with 4.5 g/ml glucose, 2 mM L-glutamine, 100 U/ml penicillin, 0.1 mg/ml streptomycin, and 10% fetal calf serum. Stable cell lines were prepared using shRNA (Gencopedia) according to manufacturer’s instructions. Cells were grown in a 10-cm culture dish, under standard conditions, at 37°C with 5% CO_2_. All cell culture materials were purchased from Biological Industries.

### Protein purification and western blot

Total proteins were extracted from the cells using a hypotonic lysis buffer (50 mM Tris-HCl, pH 7.5, 1% NP40, 150 mM NaCl, 0.1% SDS, 0.5% deoxycholic acid, 1 mM EDTA) containing protease inhibitor and phosphatase inhibitor cocktails I and II (Sigma). After 20 min centrifugation at 14,000 g at 4°C, the supernatant was collected and protein concentrations were measured using BioRad Protein Assay. Cytoplasmic and nuclear fractions were obtained using sucrose gradients as described [81]. Proteins were separated in an 8% SDS-PAGE and then electroblotted onto a Protran nitrocellulose transfer membrane (Schleicher & Schuell). Immunoblots were incubated with primary and secondary antibodies and visualized by enhanced chemiluminescence (SuperSignal West Pico chemiluminescent substrate; Thermo Scientific) and exposure to X-ray film. Data was from three separate experiments and was quantified using Image J. Error bars represent SEM.

### Co-immunoprecipitation

Cells were washed with PBS and crosslinked with 0.1% formaldehyde, and total lysates were purified and then re-suspended in immunoprecipitation (IP) buffer (50 mM HEPES [pH 7.6], 500 mM LiCl, 1 mM EDTA, 0.7% DOC, 1% NP-40, 0.1% SDS, 13 CPI). Cells were sonicated and incubated 12 h at 4°C with antibody conjugated to protein A Dynabeads (Invitrogen). Samples were washed with IP buffer. Proteins were eluted and subjected to western blot analysis. Samples were reversed crosslinked for 12 h at 55°C, and protein were eluted from the beads by adding 100 ml PBS and 20 ml SDS sample buffer (272 mM Tris-HCl [pH 6.8], 30% glycerol, 12% SDS, 20% β-mercaptoethanol, 0.01% bromophenol blue) and incubating in a thermo-shaker for 15 min at 75°C with vigorous shaking. The supernatant was moved to a new tube and boiled for 5 min at 100°C.

### Axonal transport

DRGs were dissected from E13.5 mice, followed by separation from meninges and spinal cord. Microfluidic chambers were prepared as described [68,69]. A single DRG of up to 1 mm was placed in each explant well, were allowed to adhere for 1 hour after which NGF rich media were added. On DIV 3 cells were starved for 2 hours with NGF-deprived medium, followed by addition of Quantum-Dot labeled NGF to the distal axon compartment. After 30 min incubation in 37°C, retrograde transport along the axon was imaged at 37°C and CO2 controlled environment, using Nikon Eclipse Ti microscope equipped with Yokogawa CSU X-1 spinning disc confocal. Time-lapse image analyses were carried out using Fiji and MATLAB as described [68,69].

### Wound healing assay

Cells were seeded in an ImageLock 96-well plate at a density of 27,000 cells/well and cultured until confluent. Wounds were inflicted across the cell layer using WoundMaker™. Cells were washed with PBS and supplemented with fresh medium. Selected wells were incubated with PS (final concentration 200 μg/ml), TSA (1.5 mM) or solvent. Migration was monitored using an IncuCyte Zoom microscope (Essen Bioscience), with image acquisition every 60 min for 48 h. The IncuCyte Zoom image analysis software was used to quantify wound closure.

### Nocodazole-induced microtubule depolymerisation

Cells were grown on 13-mm glass cover slips. Cells were treated with 1.0 μM nocodazole together with 200 μg/ml PS or vehicle. After 24 h, the fibroblasts were fixed for immunofluorescence study.

### Statistical analyses

All data were examined using two-tailed Student's t-test. The P-values and number of independent biological replicates (n) are indicated in the figure legends and results.

## Supporting Information

S1 Fig Generation of a conditional knockout FD mouse. (**A**) Total knockout (KO) and control mice were analyzed for DRGs size. Frozen cryo sections of E11.5 IKAP KO and control littermate embryos were immunostained for IKAP and Isl-1 as DRGs marker. Lumbar DRGs cross-sections show a decrease of DRGs size as indicated by the gross morphology and decreased number of cells expressing Isl-1. (**B**) Genotyping of *Tyrp2-Cre;IKBKAP*^FDloxP/FDloxP^ mice.
(TIF)

S2 Fig Deletion of *IKBKAP* exon did not affect TrkA subpopulation. Frozen cryo cross-sections of E13.5 CKO^*Tyrp2*^ FD and control littermate embryos were immunostained for TrkA (pink) subpopulation and DRG markers Isl-1 (green). Lumbar DRG cross-sections did not show differences in TrkA subpopulation in the DRGs of CKO^*Tyrp2*^ FD mice compared to controls. Scale bars 100 μm.
(TIF)

S3 Fig HDAC6 precipitates IKAP. HEK 293nt cell lysates immunoprecipitated with anti- HDAC6 antibody were analyzed for the indicated proteins.
(TIF)

S4 Fig Phosphatidylserine elevates acetylated α-tubulin levels and acts similarly to TSA, an HDAC inhibitor. (**A**) FD fibroblasts were immunostained for IKAP and acetylated α-tubulin following TSA or PS treatment. (**B**) Wound healing assay using HEK 293nt cells and HEK 293nt cells stably expressing shIKAP treated with TSA or PS.
(TIF)

S5 Fig Phosphatidylserine did not affect axonal transport in CKO*^Tyrp2^* FD DRGs. PS treatment did not alter NGF transport in DRG explants culture from CKO^*Tyrp2*^ FD embryos. Mean average velocities and speeds were not significant compare to vehicle treated controls.
(TIF)

S6 Fig Phosphatidylserine improves axonal transport in normal DRGs. (**A-E**) PS alters NGF transport in DRG explants culture from wild-type embryos. Labeled NGF was added to the distal side of the culture, and bright field and fluorescent images were taken 24 hours after addition of PS or vehicle. (**A**) NGF-Qdot transport was imaged in DRG neurons upon PS treatment. The arrowheads track representative faster Q-dots along the axon of PS treatment neurons. Below is a representative kymograph demonstrated faster NGF-Qdot transport of PS treated cells. (**B**) Mean average velocities and speeds (***p<0.001), (**C**) displacement, and (**D**) mean square displacement plotted vs. time of labeled NGF in wild-type DRG cultures treated with PS or vehicle. Error bars represent SEM. (**E**) Comparisons of the distribution profiles for instantaneous velocities show that PS treatment affects both the maximum velocity of NGF motility and induce an overall shift toward faster velocities.
(TIF)

S7 Fig Compare of NGF axonal transport in Heterozygotes and normal mice. The average velocities and speeds of labeled NGF were not significantly different in CKO/^*+*^ FD DRGs than control DRGs. Error bars represent ±SEM.
(TIF)

S8 Fig Phosphatidylserine rescues nocodazole treatment microtubule collapse. Normal and FD derived fibroblast were treated with nocodazole 1.0 uM (NCDZL). NCDZL collapse the microtubule network, as indicated from α-tubulin staining (green). FD fibroblasts were more susceptible to NCDZL than normal fibroblasts. Treatment with PS improved the resistance of cells to NCDZL treatment.
(TIF)

S1 Table List of primers. (DOCX)
