# Supplementary figures and images for: Phosphatidylserine Ameliorates Neurodegenerative Symptoms and Enhances Axonal Transport in a Mouse Model of Familial Dysautonomia
Source: PLoS Genet. 2016 Dec 20;12(12):e1006486. doi: 10.1371/journal.pgen.1006486 (PMC5172536; doi:10.1371/journal.pgen.1006486)

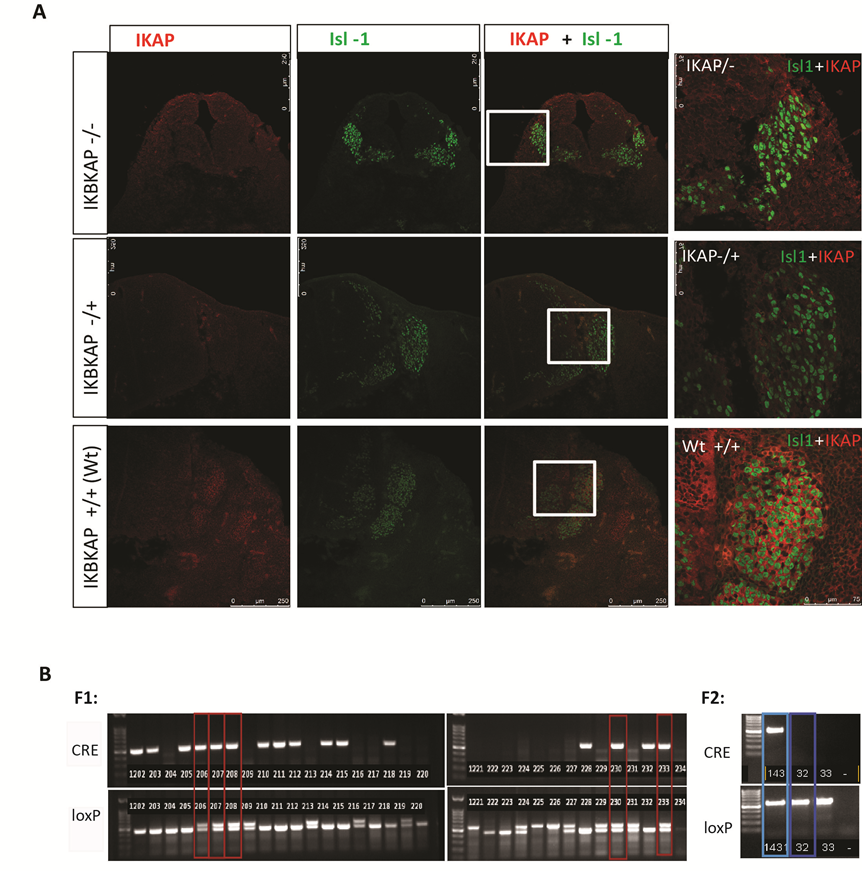

Supplement: S1 Fig — (A) Total knockout (KO) and control mice were analyzed for DRGs size. Frozen cryo sections of E11.5 IKAP KO and control littermate embryos were immunostained for IKAP and Isl-1 as DRGs marker. Lumbar DRGs cross-sections show a decrease of DRGs size as indicated by the gross morphology and decreased number of cells expressing Isl-1. (B) Genotyping of Tyrp2-Cre;IKBKAPFDloxP/FDloxP mice. (TIF) [file pgen.1006486.s001.tif]

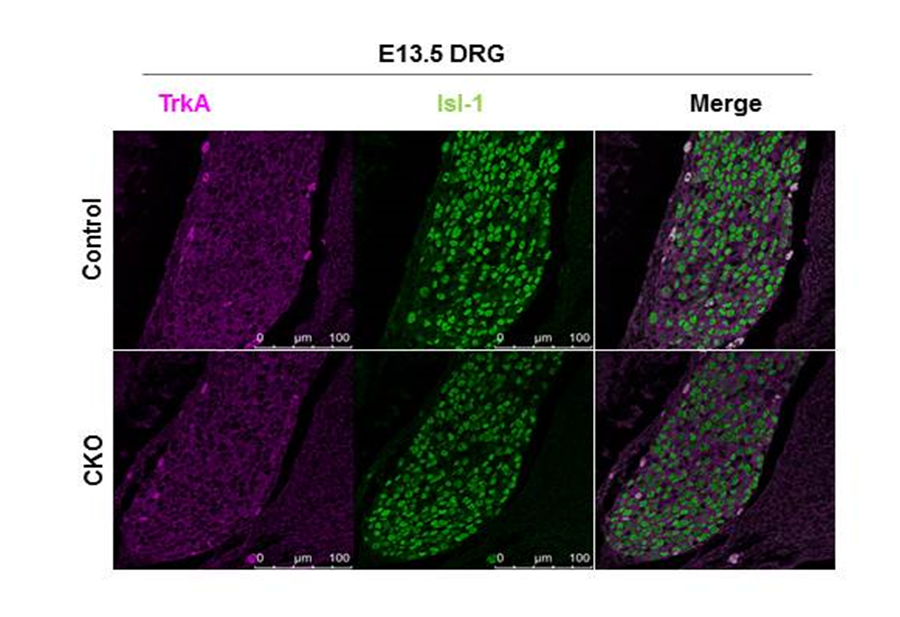

Supplement: S2 Fig — Frozen cryo cross-sections of E13.5 CKOTyrp2 FD and control littermate embryos were immunostained for TrkA (pink) subpopulation and DRG markers Isl-1 (green). Lumbar DRG cross-sections did not show differences in TrkA subpopulation in the DRGs of CKOTyrp2 FD mice compared to controls. Scale bars 100 μm. (TIF) [file pgen.1006486.s002.tif]

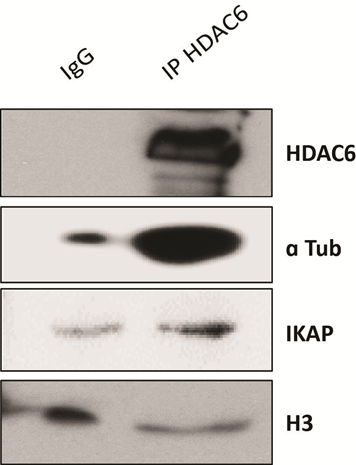

Supplement: S3 Fig — HEK 293nt cell lysates immunoprecipitated with anti- HDAC6 antibody were analyzed for the indicated proteins. (TIF) [file pgen.1006486.s003.tif]

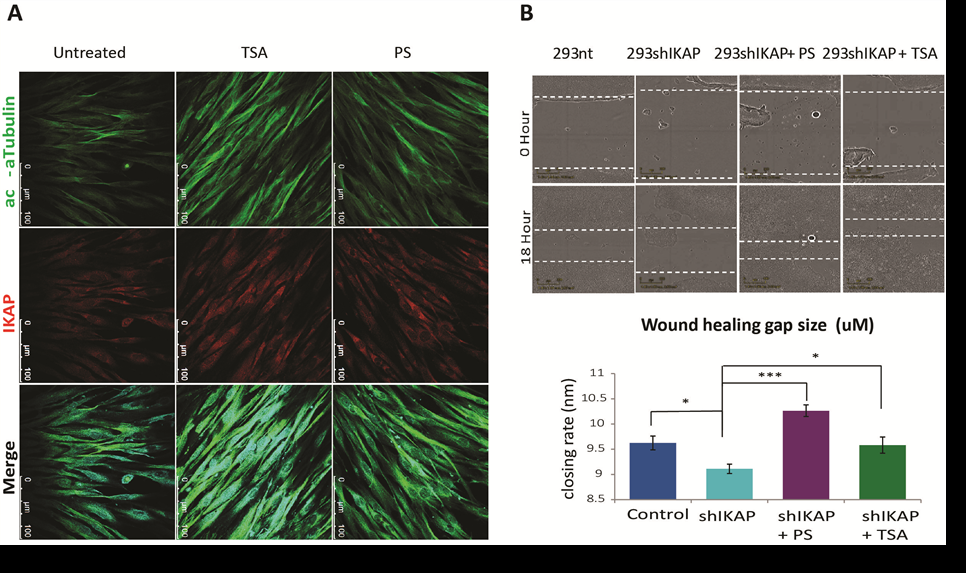

Supplement: S4 Fig — (A) FD fibroblasts were immunostained for IKAP and acetylated α-tubulin following TSA or PS treatment. (B) Wound healing assay using HEK 293nt cells and HEK 293nt cells stably expressing shIKAP treated with TSA or PS. (TIF) [file pgen.1006486.s004.tif]

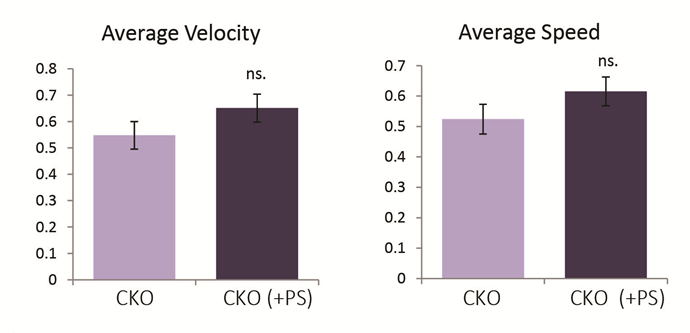

Supplement: S5 Fig — PS treatment did not alter NGF transport in DRG explants culture from CKOTyrp2 FD embryos. Mean average velocities and speeds were not significant compare to vehicle treated controls. (TIF) [file pgen.1006486.s005.tif]

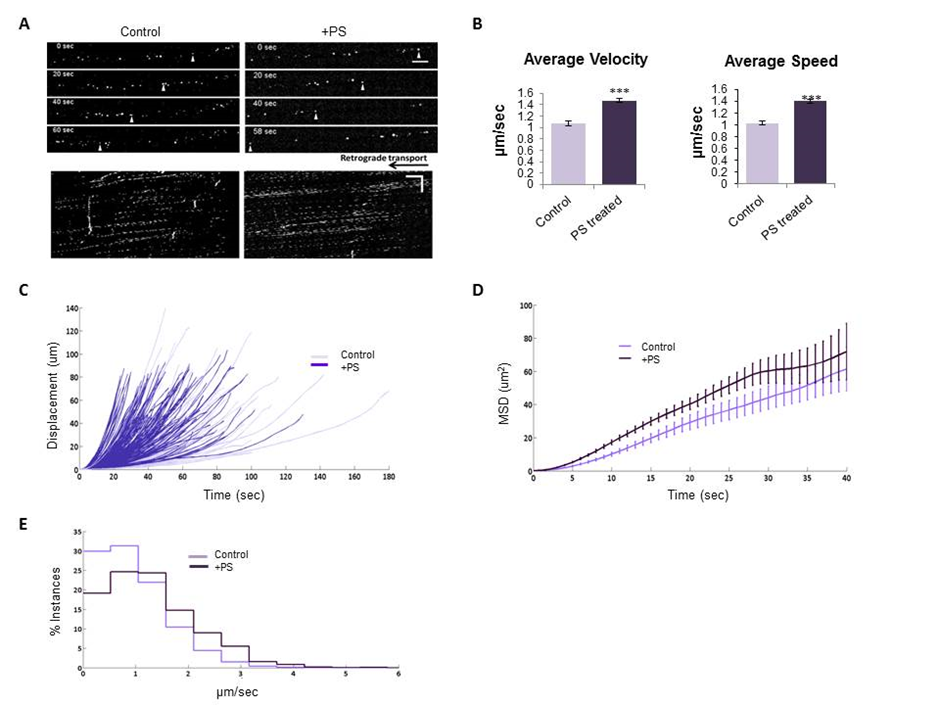

Supplement: S6 Fig — (A-E) PS alters NGF transport in DRG explants culture from wild-type embryos. Labeled NGF was added to the distal side of the culture, and bright field and fluorescent images were taken 24 hours after addition of PS or vehicle. (A) NGF-Qdot transport was imaged in DRG neurons upon PS treatment. The arrowheads track representative faster Q-dots along the axon of PS treatment neurons. Below is a representative kymograph demonstrated faster NGF-Qdot transport of PS treated cells. (B) Mean average velocities and speeds (***p<0.001), (C) displacement, and (D) mean square displacement plotted vs. time of labeled NGF in wild-type DRG cultures treated with PS or vehicle. Error bars represent SEM. (E) Comparisons of the distribution profiles for instantaneous velocities show that PS treatment affects both the maximum velocity of NGF motility and induce an overall shift toward faster velocities. (TIF) [file pgen.1006486.s006.tif]

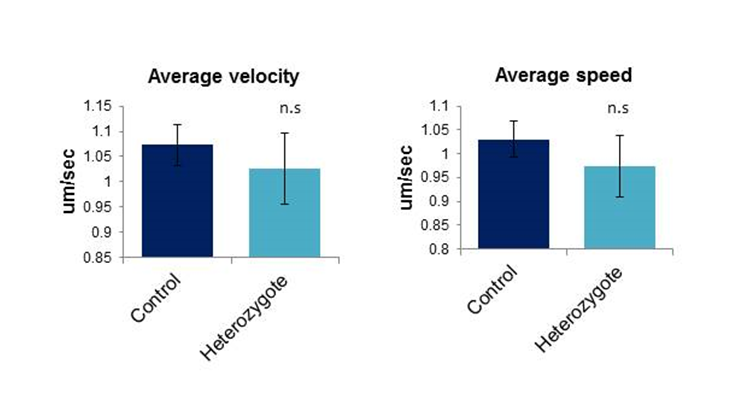

Supplement: S7 Fig — The average velocities and speeds of labeled NGF were not significantly different in CKO/+ FD DRGs than control DRGs. Error bars represent ±SEM. (TIF) [file pgen.1006486.s007.tif]

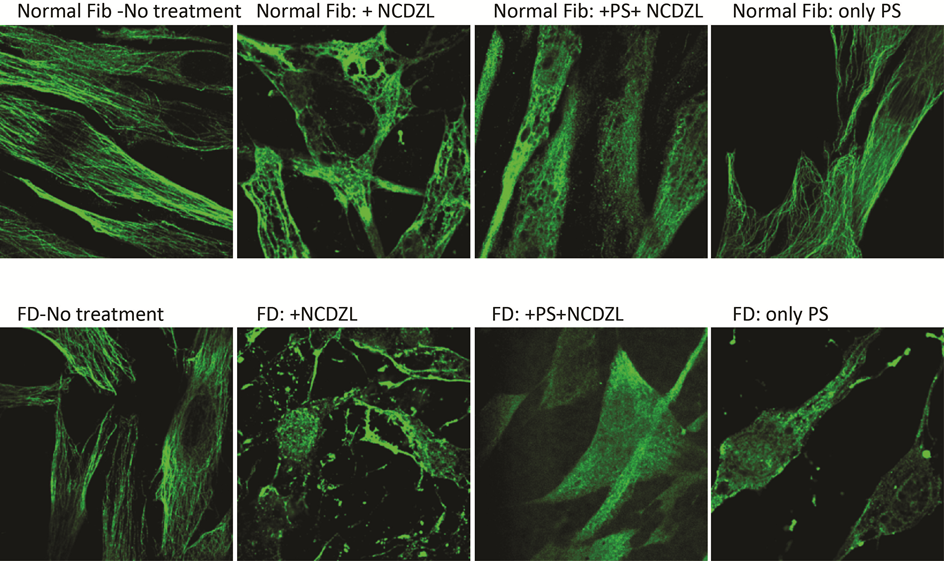

Supplement: S8 Fig — Normal and FD derived fibroblast were treated with nocodazole 1.0 uM (NCDZL). NCDZL collapse the microtubule network, as indicated from α-tubulin staining (green). FD fibroblasts were more susceptible to NCDZL than normal fibroblasts. Treatment with PS improved the resistance of cells to NCDZL treatment. (TIF) [file pgen.1006486.s008.tif]
