# Supplementary material for: Phosphatidylserine Ameliorates Neurodegenerative Symptoms and Enhances Axonal Transport in a Mouse Model of Familial Dysautonomia
Source: PLoS Genet. 2016 Dec 20;12(12):e1006486. doi: 10.1371/journal.pgen.1006486 (PMC5172536; doi:10.1371/journal.pgen.1006486)
Supplement: S1 Table — (DOCX) [file pgen.1006486.s009.docx]

**S1 Table.**

**Primers**

| **Gene** | **Forward primer** | **Revers primer** |
| --- | --- | --- |
| *MAG* | AACCTTCTGTGTTAGCGTTCC | TGATTGAGCTAGGGCTTAGTC |
| *NGF* | TAATGTCCATGTTGTTCTACACTC | TCCTTCTGGGACATTGCTATC |
| *KLK6* | TGTCAGGGTGATTCTGGAGG | GCTTCTCCTTTGATCCACAGG |
| *NCAM* | GTTTCCCTGCAGGTAGATATTG | CTGCCACTTGACACAGGAAG |
| *ELP3* | CAAGGGAGGAAGTGGATTCTC | CTGCTTGATAACGTCTCCTATA G |
| *SNCA* | TTTGTCAAGAAGGACCAGATGG | TAAGCCTCACTGCCAGGATC |
| *PPIA* | GCAGACAAAGTTCCAAAGACAG | ACCACCCTGGCACATGAATC |
| *APOD* | GGTGAAGCCAAACAGAGCAAC | GGTGGCATCAACGGGAAG |
| *REST* | GTGCGAACTCACACAGGAGAAC | TGAGTCTTCTGAGAGCTTGAGTAAGG |
| *MBP* | ATGGCTTCCTCCCAAGGC | GTG TGT GAG TCC TTG CCA G |
| *SCG10* | CGACATGGAGGTGAAGCAGAT | ATGGTGGCTTCAAGATCAGCTC |
| *TUB* | CCTGCTCATCAGCAAGATCC | TCTCATCCGTGTTCTCAACC |
| *IKAP* | TTCGGAAGTGGTTGGACAAACTT | CTTGGGGTTATGGTCATGAATC |
